# Recovery of Coastal Fauna after the 2011 Tsunami in Japan as Determined by Bimonthly Underwater Visual Censuses Conducted over Five Years

**DOI:** 10.1371/journal.pone.0168261

**Published:** 2016-12-12

**Authors:** Reiji Masuda, Makoto Hatakeyama, Katsuhide Yokoyama, Masaru Tanaka

**Affiliations:** 1 Maizuru Fisheries Research Station, Kyoto University, Nagahama, Maizuru, Kyoto, Japan; 2 Non Profit Organization Mori-umi, Nishi-moune, Karakuwa, Kesennuma, Miyagi, Japan; 3 Tokyo Metropolitan University, Minami-Osawa, Hachioji, Tokyo, Japan; 4 International Institute for Advanced Studies, Kizugawadai, Kizugawa, Kyoto, Japan; University of Hyogo, JAPAN

## Abstract

Massive tsunamis induce catastrophic disturbance in marine ecosystems, yet they can provide unique opportunities to observe the process of regeneration. Here, we report the recovery of fauna after the 2011 tsunami in northeast Japan based on underwater visual censuses performed every two months over five years. Both total fish abundance and species richness increased from the first to the second year after the tsunami followed by stabilization in the following years. Short-lived fish, such as the banded goby *Pterogobius elapoides*, were relatively abundant in the first two years, whereas long-lived species, such as the black rockfish *Sebastes cheni*, increased in the latter half of the survey period. Tropical fish species were recorded only in the second and third years after the tsunami. The body size of long-lived fish increased during the survey period resulting in a gradual increase of total fish biomass. The recovery of fish assemblages was slow at one site located in the inner bay, where the impact of the tsunami was the strongest. Apart from fish, blooms of the moon jellyfish *Aurelia* sp. occurred only in the first two years after the tsunami, whereas the abundances of sea cucumber *Apostichopus japonicus* and abalone *Haliotis discus hannai* increased after the second year. Although we lack quantitative data prior to the tsunami, we conclude that it takes approximately three years for coastal reef fish assemblages to recover from a heavy disturbance such as a tsunami and that the recovery is dependent on species-specific life span and habitat.

## Introduction

Post-disturbance recovery of flora and fauna is a major interest among community ecologists, whose specific aims include understanding diversity, stability, and productivity (e.g., [1–2]); natural succession processes [3]; and anthropogenic impacts (e.g., [4–5]) after such disturbances. Although the recovery processes should be dependent on the strength and spatiotemporal scale of the disturbance, studies have been conducted either on a small scale using intense experimental disturbances (e.g., [6–7]) or on a large scale using relatively mild natural or anthropogenic disturbances [8]. This is probably because there are limited opportunities to study the impact of large and serious disturbances except for those induced by some fishery activities (e.g., [5, 9]) or events such as oil spills [10].

A massive tsunami with a maximum run-up wave height of 40.4 m hit the Pacific coast of northeast Japan immediately after the historically large 9.0 magnitude earthquake in March 2011 [11]. A tsunami of this scale induces a thorough removal of shallow benthic fauna and vegetation as well as catastrophic sediment deposition of terrigenous clay [12]. Thus, a tsunami completely changes the habitat and causes an extremely intense disturbance to the marine ecosystem.

A tsunami can provide a unique opportunity to observe the recovery of a marine ecosystem after a heavy disturbance. Lomovasky et al. [13] studied the macrobenthic community before and after the 2007 tsunami in Peru and found an increase in the abundance of filter feeders and grazers followed by an increase in predators during the post-tsunami period. In a survey of the benthic community before and after the 2004 tsunami in Thailand [14], the presence of seagrass vegetation altered the temporal variation in macrofaunal assemblages and the subsequent recovery processes following the tsunami. The impacts of the 2011 tsunami in Japan on the intertidal flats community [15], the subtidal polychaete assemblage [16], the megabenthos assemblage in soft-bottom environments [17], and the abundance of commercially important abalone and sea urchins on rocky shores [18] have been studied. In addition, Shoji & Morimoto [19] evaluated the impact of this tsunami on the seagrass density and associated fish assemblages and revealed that fish abundance increased, whereas species richness was consistent before and after the tsunami. The abovementioned surveys were conducted at an interval of once or twice a year, except for Abe et al. [16]. However, community structure changes may occur on a shorter time scale, especially in the first year after a tsunami.

We conducted extensive surveys of the marine environment starting soon after the tsunami occurred in March 2011 at a frequency of once every two months in and around the Nishi-Moune Bay, Kesennuma, northeast Japan (Tanaka et al., unpublished data). Here, we report findings from an underwater visual census evaluation of fish and macroinvertebrate assemblages beginning two months after the tsunami and continuing for five years. In this study, following questions regarding the coastal fauna’s response to the tsunami were addressed. (1) After the tsunami, does the abundance of short-lived fish species (r-strategists) increase followed by an increase of long-lived species (K-strategists)? (2) Do long-lived species exhibit measurable growth during the first five years after the tsunami? (3) Is the fish community saturated and stabilized in the typical time span (~2–3 years) required for the reproduction of long-lived fish? (4) Do immigrant species, particularly from tropical waters, occur due to the lack of ecosystem resistance prior to the recovery? (5) Does the recovery process for the fish communities differ between locations with different levels of tsunami disturbance? In addition to fish, three invertebrate species—the moon jellyfish *Aurelia* sp., the Japanese sea cucumber *Apostichopus japonicus*, and abalone *Haliotis discus hannai*—were also evaluated. The jellyfish is an opportunistic zooplankton feeder that often increases in abundance in deteriorated environmental conditions and has a short lifespan of approximately one year [20]. In contrast, sea cucumbers are sediment feeders that digest plant-origin organic matter and are important ecological engineers with a high commercial value and a lifespan of several years [21]. Abalone is an herbivore and requires three years to mature (5 cm in shell length) and four years to recruit for fisheries (9 cm [22]). Therefore, we predicted that jellyfish would be abundant in the early stages after the tsunami, whereas sea cucumbers and abalone would require at least three years for their population to stabilize.

## Materials and Methods

**Fig 1:**
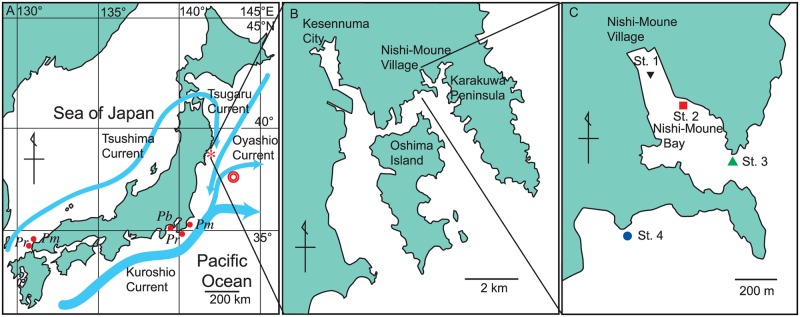
Location of post-tsunami visual censuses. (A) Map of the Japanese archipelago showing major ocean currents (blue arrows), locations of the survey sites (asterisks), and earthquake epicenter (double circle). *Pb*, *Pm*, and *Pr*, respectively, represent the previously recorded northern distribution limits of *Parupeneus barberinus*, *Parupeneus multifasciatus*, and *Plagiotremus rhinorhynchos* in the Pacific Ocean and the Sea of Japan according to Nakabo [23]. *P*. *barberinus* has never been reported in the Sea of Japan. (B) Magnified map of the survey area. (C) Four survey stations in and around the Moune Bay, Kesennuma, Miyagi Prefecture.

Underwater visual censuses were conducted every two months from May 2011 to March 2016 at four locations in and around the Nishi-Moune Bay, Kesennuma, Miyagi, Japan (38°54’N and 141°38’E) (Fig 1). No specific permission was required for underwater visual censuses in this district and the survey plan was announced to the Kesennuma Coast Guard Station and the Kessenuma Fisheries Cooperative Association. An island in the south and a peninsula in the east of the bay reduce wave energy and make this area an ideal site for culturing the oyster *Crassostrea gigas* and other bivalve species (Fig 1B). The tsunami of 2011 destroyed 44 out of 52 houses in the village located along the Nishi-Moune Bay (Fig 1C). In addition, oyster rafts were entirely wiped out, but they were rebuilt by the summer of 2012.

Four locations were selected for census evaluation based on a preliminary survey conducted in May 2011 (Table 1). Station (st.) 1 was located in the inner part of the bay where the tsunami impact was the highest having a run-up height of 15 m and estimated current velocity of 3.8 m s^-1^ [24]. The eelgrass *Zostera marina* that was abundant before the tsunami was completely eliminated after it. Station 2 was located along a shallow, rocky shore where the tsunami impact was limited. Station 3 was located at the mouth of the bay, and station 4 was outside the bay. Prior to the tsunami, st. 2 hosted a large shoal of black rockfish, *Sebastes cheni*, whereas stations 3 and 4 were good fishing grounds for the large greenling *Hexagrammos otakii* (Hatakeyama, unpublished data). In May 2011, macroalgae was found only at st. 2 where a small number of *Sargassum horneri*, partly covered by sediment, was observed.

**Table 1:** Maximum depth (m), substrate, and vegetation characteristics of each station surveyed in the first five years after the 2011 tsunami.

St	Depth	Substrate	Vegetation
1	10	Silt, sand, pebbles	Occasional *Sargassum* forest
2	6	Sand, rocky reef	Various green and brown algae and eelgrass
3	18	Silt, rocky reef	*Sargassum* and some kelp
4	20	Silt, sand, rocky reef	Various green and brown algae with some kelp

St, station

A modified line transect method termed “fin-kick transect” was employed. In this method, the distance traveled was estimated by the number of fin kicks made [25]. Through trials calibrated using a rangefinder (Nikon Laser 600, Nikon, Tokyo, Japan), it was determined that 54 fin kicks resulted in a distance of 50.1 ± 5.0 m (mean ± SD, *n* = 10) being traveled. Transect length could be either over- or underestimated with this method depending on the current, and therefore, the transects were set to counterbalance the direction (e.g., five to the north and then five to the south). Ten 50-m transects were performed at each location, and all fish within a 1-m distance from each transect were recorded; thus, ten belt transects of approximately 100 m^2^ were surveyed.

Fish species names follow Nakabo [23] and the number of individuals and body sizes of all fishes were recorded along with those of conspicuous invertebrates including the moon jellyfish, *Aurelia* sp.; the sea cucumber, *Apostichopus japonicus*; and the abalone, *Haliotis discus hannai*. Fish standard lengths were visually estimated to the nearest cm for fish <30 cm and to the nearest 5 cm for others using a ruler on an underwater slate, whereas the body lengths of sea cucumbers and shell lengths of abalone were directly measured using the ruler. Sea surface and seafloor temperatures were measured at each location. Visual surveys were conducted for approximately 30 min per station. Underwater visibility was 1–12 m with an average of 4 m at all stations.

In addition to fish abundance (the total number of fish in each transect) and species richness, total fish biomass for each transect was calculated based on the visually estimated body length of each fish species. The length of each species was converted to biomass using the length-mass relationships identified in a previous study [26] and by using parameters obtained from FishBase [27]. Abundance and biomass data were 4th root transformed prior to testing to improve homoscedasticity and also to reflect patterns of variation in less abundant species [28]. To evaluate the relative abundance of southern species, the center of distribution (COD), defined as the average latitude of the southern and northern limits of distribution in the northern hemisphere [29], was calculated for each species based on the distributions described by Nakabo [23]. A two-way factorial repeated-measures analysis of variance (rmANOVA) followed by a Holm’s pairwise test was applied to identify significant differences in fish abundance, species richness, fish biomass, and average COD for all species recorded in each transect. Year and station were included as fixed factors. Species-by-species analyses were also conducted by including the abundances of the eight most dominant fish species, moon jellyfish, sea cucumber, and abalone as response variables. Statistical comparisons of fish body length were conducted only for those species that were recorded at least three times in each year using two-way rmANOVA. Body lengths of sea cucumber and abalone were compared among years using Tukey’s honest significant difference test. Data were combined for these invertebrates for all stations due to a paucity of individuals in some years. Seafloor water temperature was also compared among years and stations using two-way rmANOVA.

Temporal shifts in fish community structure were visually inspected by ordinating Bray-Curtis similarities with non-metric multidimensional scaling (nMDS). The biomass of each species at each station was combined for each year and 4th root transformed for this analysis.

An α-level of 0.05 was considered significant except for the species-by-species analysis of abundance and body length where an α-level of 0.005 was used to control for the increased chance of Type I errors. All the analyses were conducted in R 2.15.2 [30] using the package ‘vegan’ [31].

## Results

**Fig 2:**
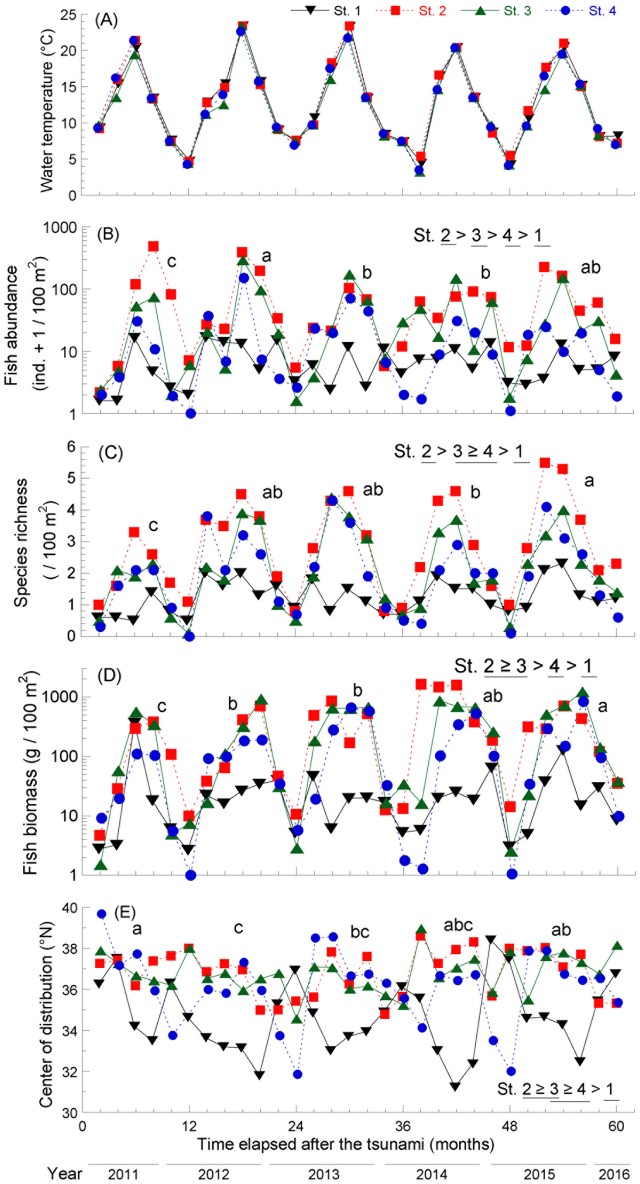
(A) Seafloor temperature, (B) fish abundance, (C) species richness, (D) fish biomass, and (E) center of distribution in the northern hemisphere of coastal fauna in the Nishi-Moune Bay in the first five years following the 2011 tsunami. Different letters indicate significant differences (*p* < 0.05) among years. Significantly different stations (st.) are underscored by different horizontal lines in the legend in each figure.

Seafloor water temperature was highest in September, ranging from 19.4°C to 23.7°C, and lowest in March, ranging from 4.1°C to 7.6°C, with the exception of the fourth year in which the lowest temperature recorded (3.2°C) was in May 2014 (Fig 2A). No significant differences were identified between locations or years in seafloor temperatures (*p* > 0.1). A total of 50 fish species from 22 families were recorded (S1 Table). Both fish abundance and species richness exhibited seasonal changes and increased with temperature. The total number of fish individuals was very low in May 2011 but rapidly increased during that year. Fish abundance differed significantly among both years (*p* < 0.01) and locations (*p* < 0.001). Abundance was significantly lower in the first year than that in all other years, and the highest value was recorded in st. 2 followed by st. 3, st. 4, and st. 1 (*p* < 0.05; Fig 2B). Species richness also differed significantly among years (*p* < 0.01) and locations (*p* < 0.001) with the lowest value recorded in the first year (*p* < 0.05; Fig 2C). Species richness was highest at st. 2 and lowest in st. 1. Total fish biomass significantly increased over the course of the study (*p* < 0.05; Fig 2D) and was highest in st. 2 and lowest in st. 1. The COD differed among years (*p* < 0.01) and locations (*p* < 0.001); it was significantly lower in the second and third years compared with the first year and was the lowest in st. 1 (*p* < 0.05; Fig 2E).

**Fig 3:**
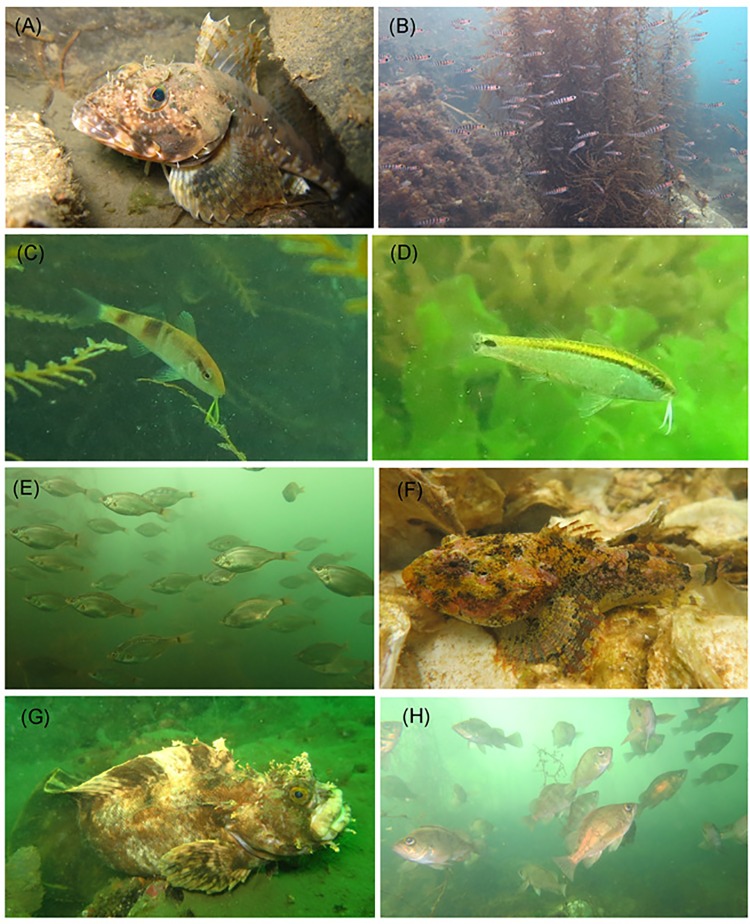
Notable fish species recorded during visual surveys in the Nishi-Moune Bay. (A) Rainbow sculpin, *Alcichthys elongatus* (Station [st.] 4 in March 2011), (B) banded goby, *Pterogobius elapoides* (st. 2 in November 2011), (C) manybar goatfish, *Parupeneus multifasciatus* (st. 3 in September 2012), (D) dash-and-dot goatfish, *Parupeneus barberinus* (st. 2 in September 2013), (E) surfperch, *Neoditrema ransonnetii* (st. 2 in July 2013), (F) great sculpin, *Myoxocephalus polyacanthocephalus* (st. 2 in May 2014), (G) fringed blenny, *Chirolophis japonicus* (st. 4 in September 2014), and (H) black rockfish, *Sebastes cheni* (st. 2 in July 2014).

Only a small number of fish (N = 41 individuals total) were found in May 2011, two months after the tsunami. The most dominant species at that time was the sunrise sculpin *Pseudoblennius cottoides* (21 individuals, 2–3 cm body length), and most of the fish observed were juveniles, except for the rainbow sculpin *Alcichthys elongatus* (8 cm body length) recorded at 14 m depth (Fig 3A). Six months after the tsunami, the banded goby *Pterogobius elapoides* was dominant and highly abundant forming a large shoal along the vegetation (Fig 3B). The vegetation was exclusively represented by *Sargassum horneri* in the first year, whereas a higher variety of macroalgae was found after the first year (R Masuda, unpublished data). In the second and third years, several warm water or tropical fish were recorded; for example, the manybar goatfish *Parupeneus multifasciatus* (Fig 3C) and the bluestriped fangblenny *Plagiotremus rhinorhynchos* were recorded in September 2012 as well as the dash-and-dot goatfish *Parupeneus barberinus* in September 2013 (Fig 3D). Relatively large individuals started to occur over the course of the study. In July 2013, shoals of the surfperch *Neoditrema ransonnetii*, containing individuals with distended bellies that were presumably gravid, were observed (Fig 3E). In the fourth year after the tsunami, cold-water origin fish such as the great sculpin, *Myoxocephalus polyacanthocephalus* (Fig 3F); the fringed blenny, *Chirolophis japonicus* (Fig 3G); and the starry flounder *Platichthys stellatus*, were recorded. Larger individuals of the black rockfish, *Sebastes cheni*, were also recorded in the fourth year (Fig 3H).

**Fig 4:**
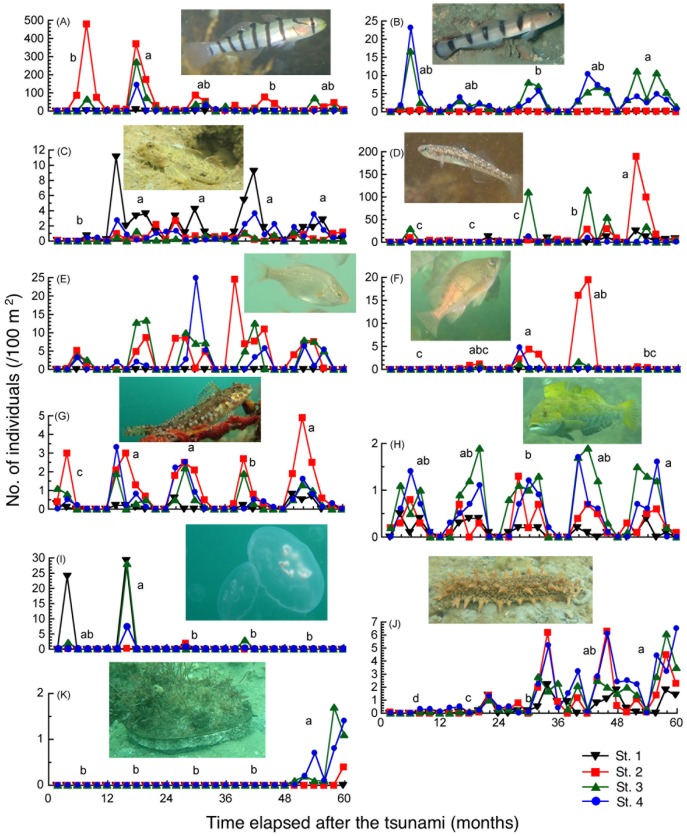
Average number of individuals across four locations in the Nishi-Moune Bay of major fish and invertebrate species over the first five years after the 2011 tsunami. (A) Banded goby, *Pterogobius elapoides*, (B) beauty goby, *Pterogobius zacalles*, (C) striped sandgoby, *Acentrogobius virgatulus*, (D) sevenspine goby, *Gymnogobius heptacanthus*, (E) surfperch, *Neoditrema ransonnetii*, (F) black rockfish, *Sebastes cheni*, (G) sunrise sculpin, *Pseudoblennius cottoides*, (H) greenling, *Hexagrammos otakii*, (I) moon jellyfish, *Aurelia* sp., (J) sea cucumber, *Apostichopus japonicus*, and (K) abalone, *Haliotis discus hannai*. Different symbols and colors correspond to stations shown in Fig 1. Different letters indicate significant differences among years.

The species-by-species analyses revealed that two species of small pelagic gobies, *Pterogobius elapoides* and *Pterogobius zacalles*, were relatively abundant in the first and second years (Fig 4A and 4B). Two epibenthic gobies, *Acentrogobius virgatulus* and *Gymnogobius heptacanthus*, tended to become abundant after the first year of the study period (Fig 4C and 4D). The surfperch, *N*. *ransonnetii*, and the black rockfish *S*. *cheni*, both having long life cycles, tended to be more abundant in the third or fourth years (Fig 4E and 4F). Two species of piscivores, the sunrise sculpin and the greenling, *Hexagrammos otakii*, gradually increased over the survey period (Fig 4G and 4H). The abundance of moon jellyfish was higher in the first two years, whereas that of sea cucumber continuously increased reaching maximum abundance in the fifth year (Fig 4I and 4J). Very few abalone were recorded in the first four years, and significantly more, mostly 9 cm in shell length or larger, were found in the fifth year (Fig 4K).

**Fig 5:**
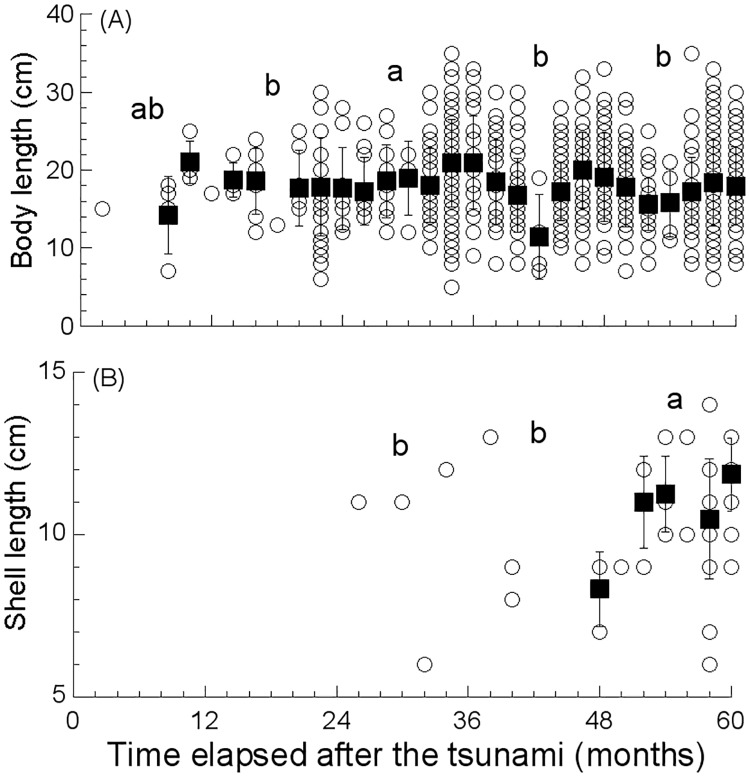
Body length of (A) sea cucumber, *Apostichopus japonicus*, and (B) abalone, *Haliotis discus hannai*, recorded in each survey across four sites in the Nishi-Moune Bay in the first five years after the 2011 tsunami. Raw data (open circles: all stations combined) and mean ± SD for each survey date (solid squares: only calculated when three or more data points were available) are shown. Different letters indicate significant differences among years at *p* < 0.05.

The body length of greenlings increased significantly for three consecutive years after the tsunami, and egg-protecting males were recorded in the fourth and fifth years at st. 3 and st. 4. The body lengths of other fish species also increased in the first two or three years (S1 Table). The sea cucumbers exhibited an increasing trend in size through the third year followed by a decline in size in the fourth and fifth years, whereas abalone size was largest in the fifth year (Fig 5).

**Fig 6:**
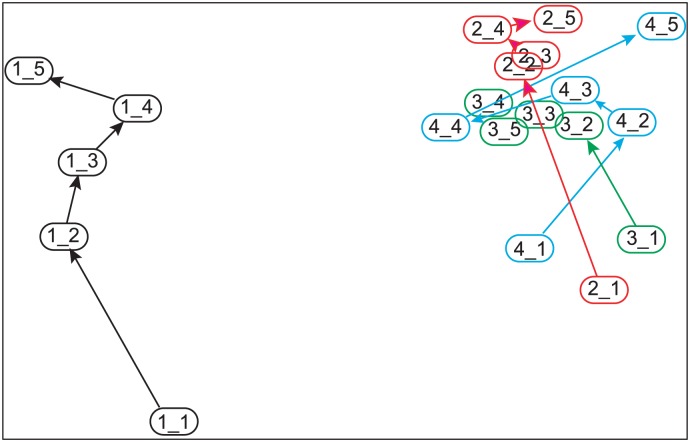
nMDS ordination plot depicting Bray-Curtis similarities of total annual biomass for each fish species observed in surveys in the Nishi-Moune Bay in the first five years post-tsunami. Each plot represents a station (st.) and year, e.g., 1_5 represents the data for St. 1 in the fifth year. The stress level was 0.14. The community structure continued to change for the entire study period at st. 1, whereas major shifts in fish communities were observed from the first to the second year only at stations 2–4.

The nMDS revealed three distinct clusters of fish assemblages: st. 1 for all five years formed one cluster; a second cluster was formed by the first year at st. 2, st. 3, and st. 4; and the third cluster was composed of the other time points and stations (i.e., the second year or later at st. 2, st. 3, or st. 4) (Fig 6). Tests for differences in similarity among these three groups supported this observation (PERMANOVA, *R*2 = 0.34, *p* = 0.001). It is worth noting that the yearly shifts in the fish assemblage at st. 1, i.e., from 1_1 to 1_5 in Fig 6, were not in the same direction as the yearly shifts observed in the other stations (shown in the upper right corner of Fig 6).

## Discussion

### Early Recovery of r-Strategists after the Tsunami

The present study supported the prediction that small-bodied opportunistic species become dominant under high disturbance conditions [32]. The banded goby, *Pterogobius elapoides*, and the beauty goby, *P*. *zacalles*, were relatively abundant in the first two years of the study. Gobies are generally short-lived, and although beauty goby can live two to three years [33], banded goby does not survive more than one year (Masuda, unpublished data based on rearing and field observations). Yamashita & Aoyama [34] conducted monthly larval fish samplings and reported that the larvae of banded goby were collected offshore in the Otsuchi Bay (ca. 50 km north from the present study site) from August to October. The lack of competitors or predators in the first year after the tsunami may have allowed a temporal population expansion of banded goby. Dominance of gobiid fish after a heavy disturbance is consistent with Desmond et al. [35] in which fish and invertebrate assemblages were monitored for 11 years in estuaries that had experienced various levels of disturbance. Our findings also coincide with Hirase et al. [36] who showed that genetic diversity was maintained after the tsunami in 2011 in the populations of intertidal goby *Chaenogobius annularis*.

Thistle [37] reviewed the succession of benthic communities after disturbances and reported that the unusually high abundance of opportunists after a disturbance might be due to both the absence of competitors and an increase in resources provided by the disturbance. The latter possibility should also be considered in the case of banded goby. Phytoplankton production was exceptionally high in the first year after the tsunami as evidenced by the elevated growth of cultured oysters, which were harvested in only seven months as opposed to the average one-and-a-half years typically required in this district (Hatakeyama, unpublished data). Yamada [38] also reported a markedly high abundance of copepods (>10 individuals L^-1^) in September 2011 in the same bay. Elevated primary and secondary production, likely due to nutrients from terrigenous and marine sediments, may have both created a suitable feeding environment for banded goby juveniles. Furthermore, the vegetation was exclusively dominated by *Sargassum horneri*, which is an annual seaweed with a long floating period from January to May [39] and a high rate of growth at 10 mm d^-1^ [40]. The tsunami in March 2011 should have provided an ideal opportunity for this alga to prevail and, thus, copious habitat for banded goby.

Striped sandgoby, *Acentrogobius virgatulus*, and sevenspine goby, *Gymnogobius heptacanthus*, were abundant in the second year or later after the tsunami. These two species were also found to be dominant in bottom trawl samples collected in this area and were highly abundant in the second year after the tsunami [41]. These species often utilize holes in the sea bottom typically made by the mud shrimp, *Upogebia major*; these holes were observed to increase in number as time elapsed after the tsunami (Masuda, unpublished data). Therefore, although they are short-lived fish species, striped sandgoby and sevenspine goby needed a relatively long period to recover due to the lack of microhabitats (i.e., holes).

Sunrise sculpin, *Pseudoblennius cottoides*, was the most dominant fish two months after the tsunami, and the individuals observed of this species were young juveniles. This fish is a small, short-lived species that matures within a year and usually dies after the first mating season [42]. The larvae of this species are common offshore of the Otsuchi Bay from November to March, whereas juveniles can be captured by a beach seine from April to August [34]. Therefore, the individuals observed in the present study were most likely in the pelagic stage when the tsunami occurred in March 2011 and thus survived.

The rainbow sculpin, *Alcichthys elongatus*, typically lives at a depth of more than 50 m but is often found in shallow waters in spring [43]. Thus, the relatively large individuals of this species may have survived after the tsunami because they were in deeper waters in March 2011. Later, in May they may have moved into shallow waters for spawning. Alternatively, they may have been passively transported to the site by the onshore currents of the tsunami.

### Fish Growth and Community Stabilization

Krishnankutty [44] predicted that post-tsunami recovery is slower in long-lived species. Indeed, the abundance of surfperch and black rockfish increased only in the latter half of the survey period. These species are both relatively long-lived and slow growing; surfperch can live for four years and attain an average standard length of 165 mm [45], and black rockfish can live at least eight years and attain an average total length of 250 mm [46]. The presence of long-lived animals increases the complexity of the fish community and thus its resistance and resilience.

Greenling is a carnivorous fish that can live for 10 years and attain an average standard length of 45 cm [47]. The abundance of greenlings showed a gradual increase throughout the survey period, and their body length steadily increased in the first three years after the tsunami followed by a plateau. The landing of this species was exceptionally high in 2015 in the Kesennuma fish market (Hatakeyama, unpublished data), although the ages of these fish were not confirmed.

Sea cucumbers, *Apostichopus japonicus*, increased in abundance throughout the survey period, reaching their maximum size three years after the tsunami. This species is an important ecological engineer that improves bottom substrate conditions and has a high commercial value [21]. Fishing of this species targeting relatively large individuals was resumed in the third year after the tsunami, which likely explains the reduction in size observed in the following years. Our results indicated that the management of sea cucumber, which is generally overexploited, might enable a full recovery through closing a fishing ground for 3 years and that rotational closure rather than size regulation might be a better management approach.

Moon jellyfish, *Aurelia* sp., were abundant in the first two years after the tsunami and then they declined. These jellyfish are adapted to turbid and hypoxic conditions and can utilize a wide range of zooplanktonic prey [48–49]. Thus, the reduction in jellyfish abundance may have reflected the recovery of oceanographic conditions after the tsunami. Furthermore, the oyster culture that resumed one-and-a-half years after the tsunami may also have facilitated the reduction of jellyfish. Oysters are highly efficient plankton feeders and indirectly compete with jellyfish that mainly feed on small zooplankton. It is also possible that the feces and pseudofeces of the cultured oysters were utilized as a food source by sea cucumbers [50].

Substantial abundance of abalone *Haliotis discus hannai* was recorded only in the fifth year. It takes 4–5 years for this species to attain 9 cm of shell length, which is the minimum size allowed to be caught in northeast Japan in this species [22]. Considering that individuals smaller than 9 cm tend to hide in cavities or behind rocks, it is likely that the population of abalone presented in this study was recruited to the district after the tsunami. This is in contrast to the case observed near the Oshika Peninsula (ca. 60 km south from the present study area) where adult abalone, although reduced in number, survived the 2011 tsunami [18]. This discrepancy is most likely due to the site-specific difference of damage induced by the tsunami; algal communities survived the tsunami in Oshika [18] whereas they were eliminated in the present study site.

### Resistance of Fish Communities to the Immigration of Tropical Vagrants

Zama [51] listed 459 fish species from 166 families historically recorded in the Miyagi Prefecture based on sampling and an extensive review of previous studies. We recorded 50 fish species, including three southern origin species—the manybar goatfish, *Parupeneus multifasciatus*; the bluestriped fangblenny, *Plagiotremus rhinorhynchos*; and the dash-and-dot goatfish, *Parupeneus barberinus*—that were absent in the above list and had been transported either from the Pacific Ocean by the Kuroshio Current or via the Sea of Japan by the Tsushima and Tsugaru Currents (Fig 1). The northern limits of distribution in the Pacific Ocean for these three species are 34°59’N– 35°08’N, whereas in the Sea of Japan the limit is approximately 34°20’N [23]. Considering the distance required to travel and the currents, the former route seems to be more likely. As our survey area was located at 38°54’N, this unusual extension of the northern limits was probably due to the lack of predators and/or competitors in the fish assemblage in Nishi-Moune Bay after the tsunami. In the fourth and fifth years after the tsunami, southern origin species were absent suggesting the recovery of a fish community composed of typical cold-temperate water species.

The average COD was significantly lower in the second and third years compared with that in the first year indicating that the earliest colonizers after the heavy disturbance were from adjacent cold-temperate waters. Later, tropical vagrants arrived to the open niches and were presumably expelled by competitors or predators in the fourth year. Station 1 was subjected to the largest tsunami impact and had the lowest value of COD. The intrusion of southern species likely occurred in locations where the level of disturbance was relatively high. These results are in agreement with those reported by Bates et al. [52] who showed that temperate reef fish communities in protected areas with high species diversity are more resistant to the colonization by warm-water species compared with those exploited by fishing pressure.

### Determinants of Ecosystem Recovery after the Tsunami

Station 1 had consistently low fish abundance and species richness throughout the study period. Prior to the tsunami, this area consisted of seagrass beds on a fine sand substrate (Hatakeyama & Tanaka, unpublished data). Whanpetch et al. [14] evaluated the effect of seagrass beds on benthic communities by conducting surveys before and after the 2004 tsunami in Thailand. They suggested that the presence of seagrass beds mitigated the impact of the tsunami [14]. The 2011 tsunami in Japan was much stronger and removed all the seagrass along with the substrate in this area. As a result, the bottom was covered by fine silt sediment and no stable vegetation had formed even five years after the tsunami. A delayed recovery in vegetation thus made the fish assemblages in this area distinct from others as depicted by the nMDS (Fig 6). It should also be noted that the substrate at st. 1 lacked a rocky reef, which may have contributed to the reduced fish species richness and abundance throughout the study period as well.

Comparisons of benthic fauna before and after the 2011 tsunami have been conducted in previous studies. Kanaya et al. [12] compared shallow lagoon macrozoobenthic assemblages prior to the tsunami (2005–2008) with those in 2011 and found an increase in the density of benthic fauna particularly for opportunistic taxa, and a decline in species richness. Benthic assemblages collected at 22 m depth in the innermost part of Onagawa Bay, Miyagi, also showed a remarkable increase of opportunistic polychaetes as pioneer species after the tsunami and a concurrent decrease of those having no planktonic larval phase [16]. Seike et al. [17] compared megabenthos assemblages in soft bottom environments in 2010, 2011, and 2012 and revealed a significant change in fauna after the tsunami including the disappearance of the sand dollar, *Scaphechinus mirabilis*, and the appearance of the heart urchin, *Echinocardium cordatum*. Although we lack quantitative data on the fauna of the survey sites before the tsunami, we frequently captured black rockfish at st. 2 and greenlings at st. 3 and st. 4 prior to the tsunami in hook-and-line fishing (Hatakeyama & Tanaka, unpublished data). Therefore, the combined qualitative and quantitative data indicated that this fauna was largely eliminated by the tsunami and followed by a recolonization first by short-lived r-strategists and then by larger, longer-lived species similar to what was found by Seike et al. [17]. Nevertheless Japanese smelt, *Hypomesus japonicus*, a cold-temperate species with its southern distribution limit in northeast Japan [23], had frequently been captured in this area before the tsunami (Ogata, unpublished data) but was not encountered in any of our visual censuses. Thus, the lack of quantitative data before the tsunami leaves the possibility that the regenerated fish assemblages might be distinct from the pre-tsunami forms.

The observed regeneration process of fauna after a tsunami contrasts with the findings of Lefèvre and Bellwood [6] who reported that after the removal of a coral reef fish community using clove oil, larger species increased earlier than smaller ones. They suggested that larger and more mobile species recolonized from nearby reefs whereas smaller species relied on recruitment from planktonic larvae [6]. In contrast to this experimental removal, a tsunami disturbance is widespread such that recolonization from adjacent areas may be less likely to happen. Similar to the results of the coral reef experiment, rapid regeneration of a fish assemblage in an estuary that had experienced hypoxia and where reinvasion was possible from adjacent areas has been reported [53].

Halford et al. [54] studied the recovery of coral reefs and reef fish communities after a heavy storm and found that fish communities needed approximately 10 years to reach the pre-storm levels, following the recovery of the coral reefs. Emslie et al. [55] found that sites with relatively high topographic complexity required less recovery time, suggesting that the recovery process is dependent on substrate structure. In the case of the temperate reef fish and invertebrate communities, their major habitat consists of macroalgal forests with a turnover rate of one to four years. Therefore, a recovery time of three years for the fish assemblage in our study site was within the predicted range.

### Constraints of Visual Census and Perspectives

Underwater visual censuses generally underestimate fish abundance and species richness due to the inability of observers to find cryptic, nocturnal, or highly mobile species [56]. Nakayama et al. [41] collected a substantial number of flatfish juveniles in this area using a bottom trawl, whereas conger eel, *Conger myriaster*; Japanese eel, *Anguilla japonica*; Japanese seabass, *Lateolabrax japonicus*; and chub mackerel, *Scomber japonicus* were captured using other methods (Hatakeyama et al., unpublished data). These fish were either few in number or entirely absent in our visual census (S1 Table). Therefore, although visual censuses are an efficient means to evaluate temporal variation in fish assemblages, particularly as measured by biomass [56], the combination of this method with others is required for a better understanding of community structure. The use of a video camera (e.g., [57]) or environmental DNA method (e.g., [58]) is promising, especially with respect to non-invasive species detection.

Typical temperate coastal reef fish assemblages in Japan show regular seasonal fluctuations both in abundance and in species richness [29]. Fish assemblages in the present study sites had stabilized two years after the tsunami, and the recovery of long-lived animals was evident in the third year. Therefore, we estimate that the average recovery time after the tsunami is approximately three years. However, fish assemblages generally exhibit some annual fluctuation depending on oceanographic conditions and interannual fluctuations of abundances are species-specific [29]. Thus, further observations are required to gain more insight into the succession process and resilience of coastal reef fauna in cold-temperate waters.

## Supporting Information

S1 Table Summary of fishes encountered in bimonthly censuses at four locations in the Nishi-Moune Bay over five years with the number of individuals and mean body lengths for each year. Lowercase letters represent significant differences in mean body length among years.
(XLS)

S2 Table Seafloor water temperature of each station in the Nishi-Moune Bay during bimonthly surveys over the course of five years. (XLS)

S3 Table Abundance, average body length, and estimated mass of each fish species recorded in each transect at four locations in the Nishi-Moune Bay during bimonthly surveys over the course of five years. (XLS)

S4 Table Parameters used to calculate fish body mass and center of distribution in the northern hemisphere for each species observed in bimonthly surveys in the Nishi-Moune Bay over a five-year period. (XLS)

S5 Table Body length of sea cucumber *Apostichopus japonicus* recorded in each transect at four locations in the Nishi-Moune Bay during bimonthly surveys over the course of five years. (XLS)

S6 Table Shell length of abalone *Haliotis discus hannai* recorded in each transect at four locations in the Nishi-Moune Bay during bimonthly surveys over the course of five years. (XLS)
